# Multidisciplinary prospective study of standardized pelvic lymph-node dissection focusing on the dorsal obturator nerve region

**DOI:** 10.1007/s10147-026-02993-5

**Published:** 2026-02-24

**Authors:** Tomokazu Sazuka, Yuji Habu, Keisuke Matsusaka, Tetsuro Maruyama, Takayuki Arai, Hiroaki Sato, Keisuke Ando, Manato Kanesaka, Shinpei Saito, Sangjon Pae, Yusuke Imamura, Hirokazu Usui, Ayumu Matsuoka, Natsuko Nakamura, Rie Okuya, Nozomi Sakai, Eri Katayama, Toru Tochigi, Atsushi Hirata, Takeshi Sugawara, Jun-ichiro Ikeda, Gaku Ohira, Kaori Koga, Shinichi Sakamoto

**Affiliations:** 1 Department of Urology, Chiba University Graduate School of Medicine, 1-8-1 Inohana, Chuo-ku, Chiba, 260-8670 Japan; 2 Department of Obstetrics and Gynecology, Reproductive Medicine, Chiba University Graduate School of Medicine, Chiba, Japan; 3 Department of Diagnostic Pathology, Chiba University Graduate School of Medicine, Chiba, Japan; 4 Department of Frontier Surgery, Chiba University Graduate School of Medicine, Chiba, Japan; 5 Clinical Research Center, Chiba University Hospital, Chiba, Japan

**Keywords:** Pelvic lymph node, Cancer, Multidisciplinary, Lymph-node dissection, Prospective, Obturator lymph node

## Abstract

**Background:**

Pelvic lymph-node dissection is performed across multiple surgical specialties. However, inconsistent terminology and unclear anatomical boundaries hinder standardization. This study established a multidisciplinary team with a shared anatomical understanding, with the aim to prospectively evaluate standardized pelvic lymph-node dissection, focusing on the dorsal region of the obturator nerve.

**Methods:**

A prospective observational study was conducted at a single institution from November 2022 to 2025. A team of urology, gastrointestinal surgery, gynecology, and pathology specialists received standardized anatomical training. A total of 106 patients undergoing pelvic lymph-node dissection for pelvic cancers were enrolled. Pelvic regions were predefined into seven areas. Data on surgical outcomes, lymph-node yield, complications, operative time, and quality of life were collected. Central pathology review and photographic scoring were performed.

**Results:**

Over 90% of surgeons rated the anatomical classification as clear. Planned lymphadenectomy was completed in over 95% of cases. The obturator region was consistently dissected. Dissection of the dorsal obturator nerve region did not increase the number of retrieved or positive lymph nodes but extended operative time by 15 min per side. No lymphadenectomy-related complications were observed in 96% of patients. Quality of life declined at 1 week postoperatively and stabilized by 1 month.

**Conclusions:**

A multidisciplinary, standardized approach to pelvic lymph-node dissection is feasible and facilitates implementation across specialties. Dissection of the dorsal obturator nerve region increases operative time without demonstrating additional oncological advantage in this study. Standardized anatomical frameworks may facilitate safer and more consistent practice.

## Introduction

Pelvic surgery spans across multiple disciplines, including gastrointestinal surgery, gynecology, and urology, and pelvic lymph-node dissection is commonly performed in all these fields. In recent years, pelvic lymph-node regions have been jointly defined in Japan by pelvic surgical departments [1–3]. The use of clear anatomical boundaries, particularly for the ureterohypogastric nerve fascia and the vesicohypogastric fascia, has made previously ambiguous borders more clearly distinguishable. Historically, the lymph-node regions were most commonly classified following the IJCO 2003 classification [4]. However, this classification posed challenges, especially regarding the delineation of the lateral area of the internal iliac artery. Consequently, variations in the terminology used to describe target lymph nodes were observed across institutions and among individual clinicians. The widespread adoption of laparoscopic and robotic surgery, along with the ability to share surgical images, has rapidly accelerated.

To establish a shared understanding of standardized pelvic anatomy among surgeons who frequently perform pelvic-related procedures, we established a cross-sectional institutional team. We conducted a Prospective Study on Pelvic Lymph node dissection with the aim of generating foundational data to address the unresolved issue of the need for standardized understanding of pelvic lymph-node regions. (PSPL) To the best of our knowledge, there have been no reports of the establishment of a multidisciplinary team across departments within a single institution to conduct a prospective observational study on pelvic lymph-node dissection. In this initial report, we present the formation of such a team, the design and implementation of the prospective observational study, and findings of obturator lymph-node dissection focusing on the dorsal region of the obturator nerve.

## Patients and methods

This study was approved by the Institutional Review Board of Chiba University (approval number, M10444) and performed in accordance with the principles outlined in the 1964 Declaration of Helsinki and its later amendments. We obtained written informed consent from all patients before surgery.

**Fig. 1:**
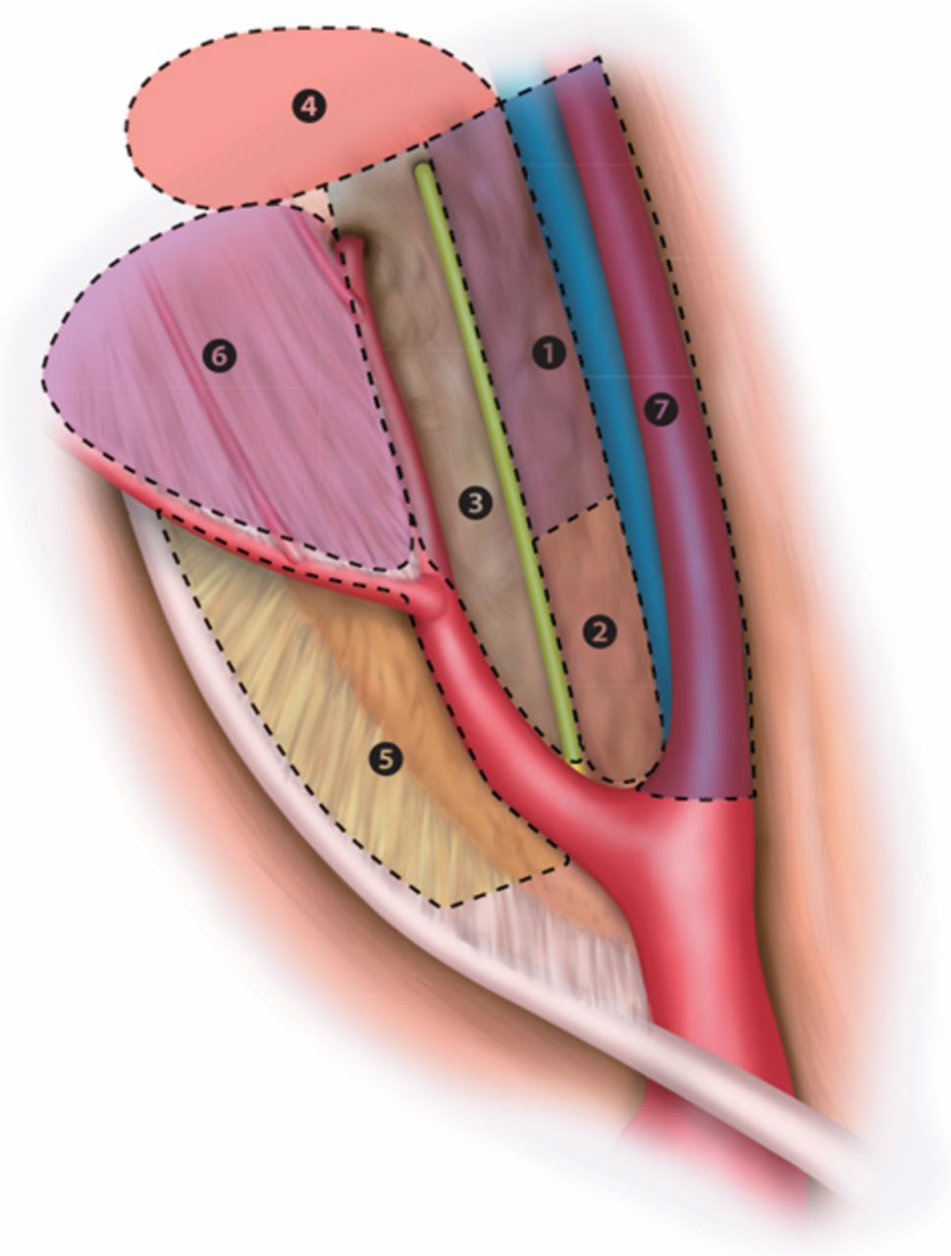
A schematic of the pelvic cavity divided into seven regions as defined in the PSPL study

This study included all physicians from the departments of urology, gastrointestinal surgery, gynecology, and pathology at Chiba University Graduate School of Medicine (Chiba, Japan). We used videos and photographs to explain the anatomical terminology and lymph-node regions related to pelvic lymphadenectomy, as defined by the core members of the study (the study authors, including six lead physicians, with two from each clinical department) (Table 1, Fig. 1). Following the instructional session, a test was conducted to ensure standardized understanding of the lymph-node regions among participants. To facilitate anatomical recognition during the study period, photographs and videos were available in the hospital’s electronic medical record system.

**Table 1:** Definition of pelvic lymph-node areas in the PSPL study

	Cranial	Caudal	Ventral	Dorsal	Internal	External
Obturator LN	Bifurcation of internal and external iliac artery	Arcus tendinous levator ani	Dorsal aspect of the external iliac vessels	Dorsal aspect of the obturator internus muscle and the vesicohypogastric fascia	Ventral aspect of the internal iliac artery and vesicohypogastric fascia	Psoas major muscle, obturator internus muscle, and levator ani muscle
1	Umbilical ligament base	Deep femoral circumflex vein (pelvic wall)	Dorsal aspect of the external iliac vessels	Obturator nerve	Ventral aspect of vesicohypogastric fascia	Psoas major muscle, obturator internus muscle
2	Bifurcation of internal and external iliac artery and vein	Umbilical ligament base	Dorsal aspect of the external iliac vessels	Internal iliac artery	Internal iliac artery	Psoas major muscle, obturator internus muscle
3	Umbilical ligament base	Obturator foramen	Obturator nerve	Dorsal aspect of the obturator internus muscle and the vesicohypogastric fascia	Ventral aspect of vesicohypogastric fascia	Psoas major muscle, obturator internus muscle
4	Obturator foramen	Arcus tendineus levator ani	Arcus tendineus levator ani	Dorsal aspect of the vesicohypogastric fascia	Ventral aspect of vesicohypogastric fascia	Levator ani muscle
Internal iliac LN	Bifurcation of internal and external iliac artery	Alcock’s canal	Dorsal aspect of the vesicohypogastric fascia and internal iliac artery	Sciatic nerve, ventral pelvic floor	Ureterohypogastric nerve-fascia	Internal iliac artery
5	Bifurcation of internal and external iliac artery	Umbilical ligament	Internal iliac artery	Sciatic nerve, ventral pelvic floor	Ureterohypogastric nerve-fascia	Internal iliac artery
6	Umbilical ligament base	Alcock’s canal	Dorsal aspect of the vesicohypogastric fascia	Sciatic nerve, ventral pelvic floor	Ureterohypogastric nerve-fascia	Internal iliac artery
External iliac LN	Bifurcation of internal and external iliac artery	Deep femoral circumflex vein (pelvic wall)	Accessory pudendal nerve	External iliac vein	External iliac artery and vein	Accessory pudendal nerve
7	Bifurcation of internal and external iliac artery	Deep femoral circumflex vein (pelvic wall)	Accessory pudendal nerve	External iliac vein	External iliac artery and vein	Accessory pudendal nerve

*PSPL* Prospective Study on Pelvic Lymph-node dissection, *LN* lymph node

This study included patients who received pelvic lymph-node dissection and primary site resection for pelvic organ cancer between November 2022 and 2025 in Chiba University Hospital. We prospectively collected data for 106 patients. Patient data at the preoperative stage included age, sex, height, weight, body mass index (BMI), primary site, clinical stage, preoperative treatment, total protein (g/dL), albumin (g/dL), history, medication, planned lymph-node dissection area (selected from 1–7, Table 1), ICIQ-SF score, and FACT-G7 score. Data at postoperative week one included type of surgical approach, sequence of lymphadenectomy and primary tumor removal, total operative time, pelvic lymph-node dissection time (separately for left and right sides), total blood loss, use of blood products, method of lymphatic vessel ligation, use of hemostatic agents at dissection sites, maximum drain output, total protein (g/dL), albumin (g/dL), intraoperative complications, clarity of the definition of the dissection area, actual dissection area (selected from 1–7, Table 1), ICIQ-SF score, and FACT-G7 score. Data at postoperative 1 month included number of resected and positive lymph nodes in the internal iliac, external iliac, and obturator regions, separately for left and right sides, total number of resected lymph nodes and number of positive lymph nodes in the entire surgery, pathological diagnosis of the primary tumor, postoperative complications, total protein (g/dL), albumin (g/dL), presence or absence of postoperative adjuvant therapy, ICIQ-SF score, and FACT-G7 score.

In this study, the lymph nodes were removed, separated by the surgeon, and submitted to pathology, taking into consideration that excess fat can make lymph-node evaluation difficult. The submitted lymph nodes were evaluated by a central pathologist (K.M.) to minimize variability in evaluation.

**Fig. 2:**
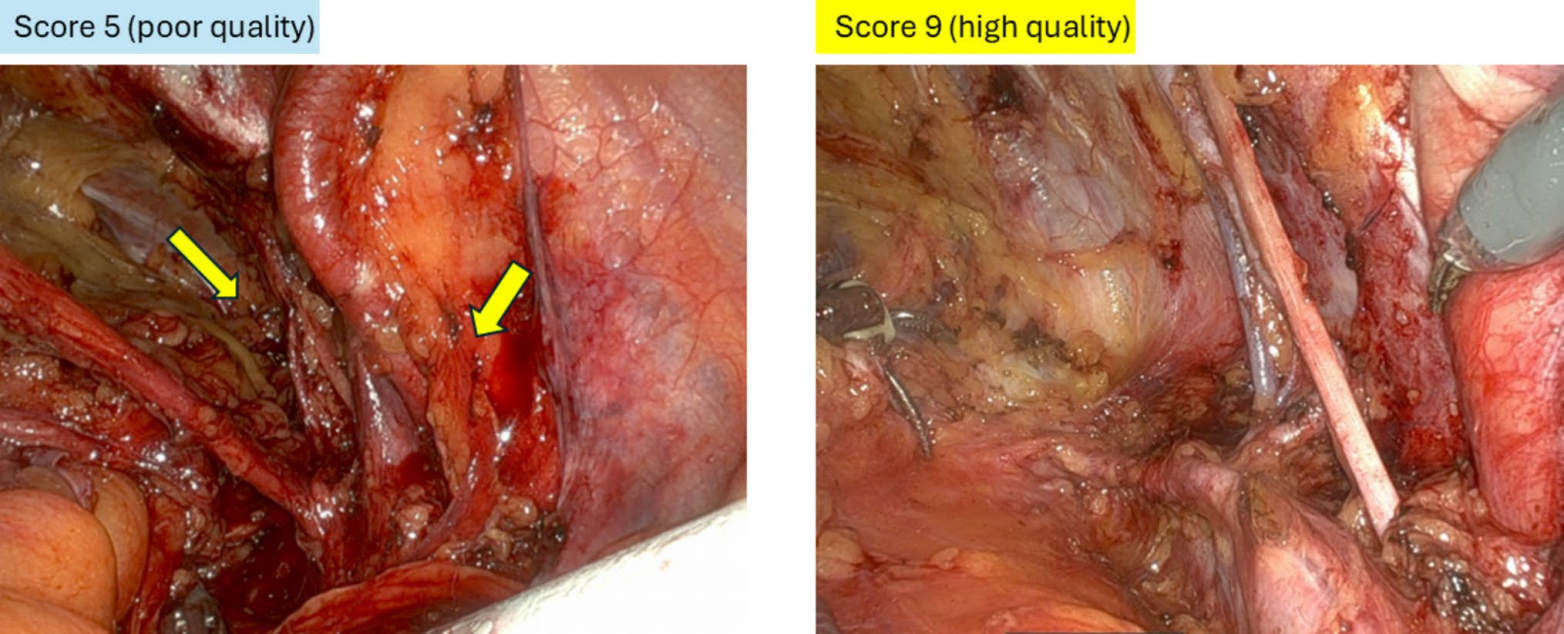
Scoring evaluation of lymph-node dissection using intraoperative photographs. Scores ranged from 0 to 10, with a passing score defined as 6 points. The median score among the six evaluators was adopted. Representative images of poor and high-quality dissections in right pelvis are shown. In the left panel, the obturator nerve and external iliac vessels were not properly exposed

We, the core members of this project (including six lead physicians, with two from each clinical department), conducted a scoring evaluation of the lymph-node dissection using intraoperative photographs. Scores ranged from 0 to 10, with a passing score defined as 6 points. The median score among the six evaluators was used for analyses. The evaluations were conducted individually for the specific regions that were dissected (Table 1 and Fig. 1) and the overall area of dissection (Fig. 2).

Complications were diagnosed using the Common Terminology Criteria for Adverse Events version 5.0. Statistical analyses were performed using JMP Pro 18.0.1 (SAS Institute Inc., Cary, NC, USA), and two-sided *P* < 0.05 denoted statistical significance. Intergroup comparisons were performed using the Mann–Whitney *U* test and *χ*^2^ test.

## Results

### Patient characteristics

The characteristics of the 106 patients with pelvic cancer enrolled in the study are presented in Table 2. The median patient age was 67 years, and 51.9% of the patients were male. The primary lesion location was bladder in 48 patients, uterus in 27, prostate in 17, rectum in 3, and unknown in 1. Clinically positive lymph nodes were detected in 10 patients (9.4%). Among the 106 patients, 47 (44.3%) received preoperative systemic treatment and/or radiation. In the overall group, 67 (63.2%) underwent robotic surgeries and 39 (36.8%) underwent open laparotomy. Lymph-node dissection followed by primary site resection was performed in 74 (69.8%) patients, and primary site resection followed by lymph-node dissection was performed in 32 (30.2%) patients.

**Table 2:** Patient characteristics (*n* = 106)

Characteristic	
Age, years, mean (range)	67 (27–84)
Sex, *n* (%)	
Male	55 (51.9)
Female	51 (48.1)
BMI, kg/m^2^ (range)	23.7 (12.2–39.4)
Primary lesion site, *n* (%)	
Bladder (including ureter, urethra)	48 (45.3)
Uterus	27 (25.5)
Prostate	17 (16.0)
Rectum	3 (2.8)
Unknown primary cancer	1 (0.9)
Clinically positive lymph node	10 (9.4)
Preoperative treatment, *n* (%)	47 (44.3)
Surgical procedure, *n* (%)	
Robot	67 (63.2)
Open	39 (36.8)
Removal, *n* (%)	
LND 1st	74 (69.8)
Primary site resection 1st	32 (30.2)

*BMI* body mass index, *LND* lymph-node dissection

### Lymph-node dissection

All physicians underwent training or education on the defined areas before surgery. The surgeons performed the surgical procedures and were subsequently assessed on their understanding of the classification method. More than 90% of the surgeons rated the PSPL classification as “clear” or better.

The surgeons preoperatively designated the pelvic regions to be dissected, classified as areas 1 through 7 (Table 1, Fig. 1), and then proceeded with the surgical procedures. In four cases, severe adhesions from previous surgeries prevented the planned lymphadenectomy. In one case, the planned dissection could not be performed because of advanced cancer progression. In over 95% of cases, the planned lymphadenectomy was successfully completed as scheduled.

After surgical field exposure, the median time required for unilateral lymphadenectomy was 44 min on the right side and 41 min on the left side. The median number of lymph nodes removed on the right and left sides was 12. Positive lymph nodes were identified on the right side in 11 cases and positive nodes were identified on the left side in 6 cases.

Intraoperative complications during lymphadenectomy included venous bleeding in three cases and sciatic nerve injury in one case. No complications related to lymphadenectomy were observed in 96% of the cases.

**Fig. 3:**
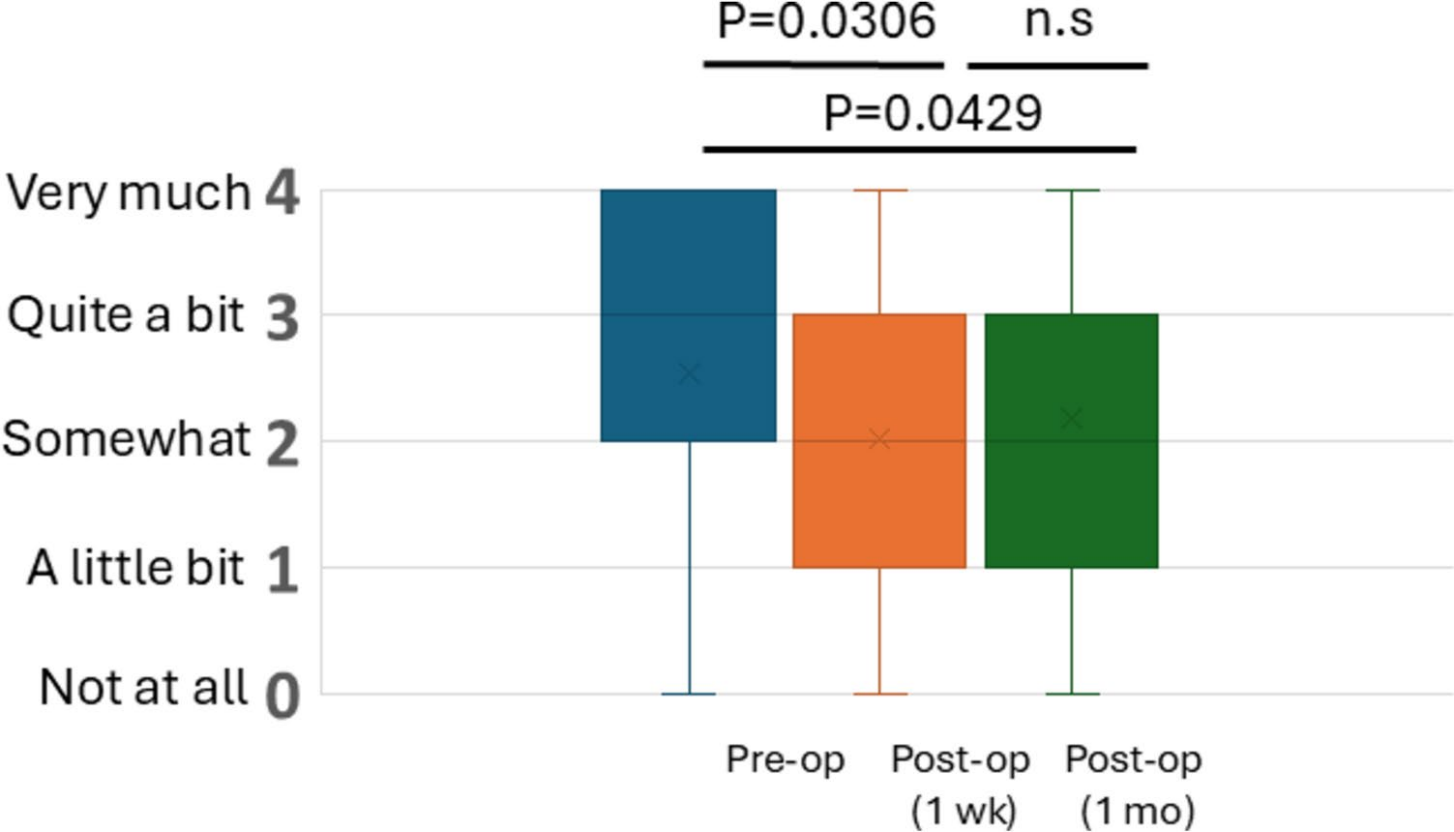
Changes in quality of life as assessed by GF7 of the FACT-G7. Distributions of responses to the statement “I am content with the quality of my life right now” are shown

Changes in quality of life (QOL) were assessed by the FACT-G7 questionnaire at three time points: preoperatively, 1 week postoperatively, and 1 month postoperatively (Fig. 3). QOL significantly decreased from the preoperative period to 1 week and 1 month after surgery. (*p* = 0.0306, *p* = 0.0429, respectively) No significant difference in QOL was observed between 1 week and 1 month postoperatively.

### Actual lymph-node dissection sites using PSPL criteria

Table 3 shows the lymph-node dissection sites from the surgeries performed in this study across cancer types. The obturator lymph-node region was the only area consistently dissected across all cancer types. The bladder and prostate generally shared the same lymph-node dissection area. All pelvic area were dissected. In uterine cancer, dissection was performed in the external iliac region, while the internal iliac region was generally not dissected. In contrast, rectal cancer involved dissection of the internal iliac region but not the external iliac region. A more detailed analysis revealed that areas 1 and 2 in the obturator region were almost always dissected. Area 3, corresponding to the dorsal side of the obturator nerve, was dissected in 82 patients on each side. Area 4 was the least frequently dissected site among all subregions of the obturator area. In the internal iliac region, area 6 (caudal) was dissected more frequently than area 5 (cranial).

**Table 3:** Actual lymph-node dissection sites using PSPL criteria

		Total	Bladder (*N* = 48)	Uterus (*N* = 27)	Prostate (*N* = 17)	Rectum (*N* = 3)	Unknown site (*N* = 1)
*Right*							
Obturator	1	89	44	26	16	2	1
	2	95	48	27	17	2	1
	3	72	45	8	16	2	1
	4	65	44	2	16	2	1
Internal	5	65	45	2	15	2	1
	6	78	45	14	16	2	1
External	7	88	45	26	16	0	1
*Left*							
Obturator	1	90	45	25	16	3	1
	2	89	45	24	16	3	1
	3	72	45	7	16	3	1
	4	66	39	8	16	2	1
Internal	5	65	38	8	16	2	1
	6	77	44	14	16	2	1
External	7	88	46	25	16	0	1

*PSPL* prospective study on Pelvic Lymph-node dissection

### Details of the obturator area

**Fig. 4:**
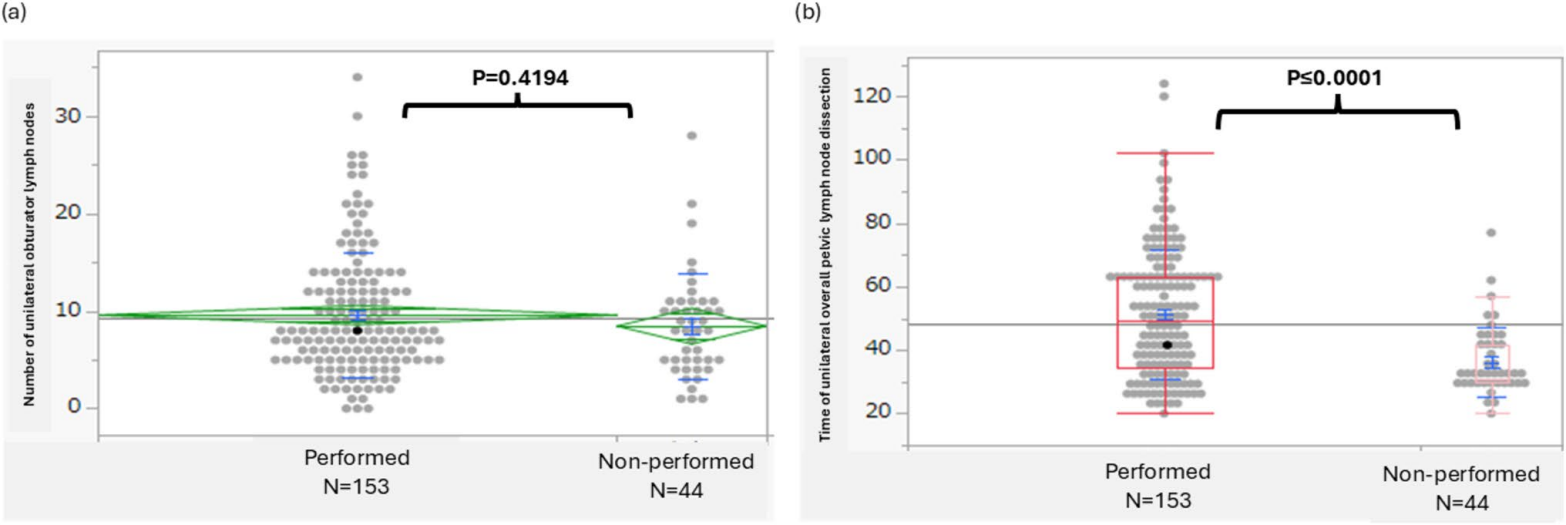
Number of unilateral obturator lymph nodes and dissection time by the dorsal area of obturator nerve dissection (PSPL-3 area). **a** Number of resected unilateral obturator lymph nodes; **b** time of unilateral overall pelvic lymph-node dissection

**Fig. 5:**
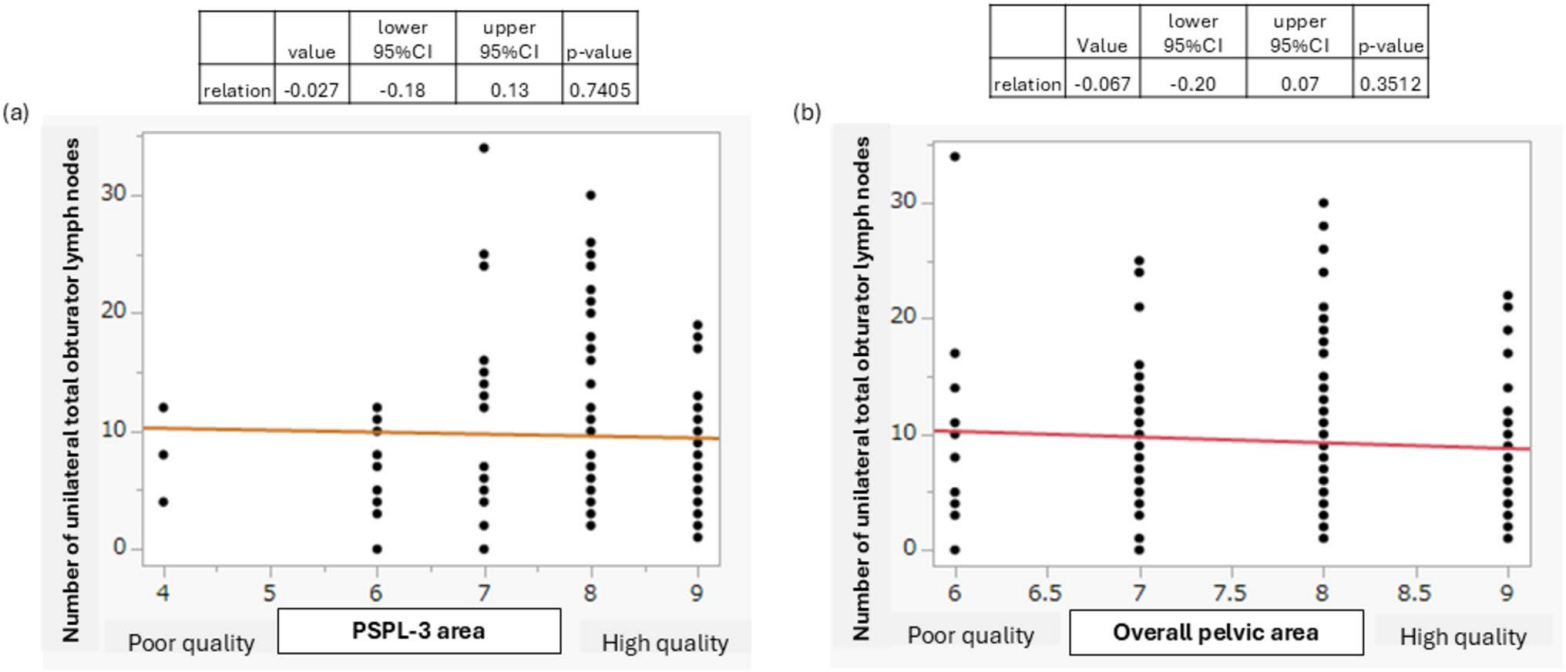
Relationship between photographic scoring and dissected lymph-node number of the PSPL-3 area and unilateral overall pelvic area. **a** Relation between PSPL-3 area and number of unilateral total obturator lymph nodes. **b** Relation between unilateral overall pelvic area and number of unilateral total obturator lymph nodes

Among the various cancer types, the obturator region was the only area consistently dissected (Table 3). We focused on the dorsal region PSPL-3 of the obturator nerve and conducted an analysis comparing cases with and without lymphadenectomy performed in this region. The mean number of removed obturator lymph nodes was 9.6 in the group that underwent dissection in this region compared with 8.5 in the group without dissection; no significant difference was observed between the two groups (Fig. 4a). No significant difference was observed in the positive detection rate between the two groups. Inclusion of region 3 in lymphadenectomy resulted in a 15-min prolongation of operative time per side, with a statistically significant difference (Fig. 4b). No significant difference was observed in the complication rate associated with lymphadenectomy including region 3 as data not shown. In the photographic assessment focusing solely on region 3, no significant difference was observed in the number of removed obturator lymph nodes by the evaluation (*p* = 0.7405) (Fig. 5a). Similarly, when assessing the overall unilateral dissection, no correlation was found between the photographic evaluation and the number of lymph nodes removed (*p* = 0.3512) (Fig. 5b).

## Discussion

We established a pelvic lymphadenectomy team comprising specialists from three pelvic clinical departments and the Department of Pathology. We conducted a prospective observational study on pelvic lymph-node dissection under a unified anatomical understanding through prospective registration. On the basis of previously published studies [1–3], we divided the pelvic region into seven anatomical subregions to better reflect the actual practice of lymphadenectomy. (Table 1, Fig. 1) We evaluated the real-world practice of pelvic lymph-node dissection, including its implementation status, associated complications, dissection areas, number of resected lymph nodes, number of positive nodes, the time required for the procedure, and change of QOL. Furthermore, we elucidated the detailed characteristics of the obturator lymph-node region, which is commonly dissected in urology, gastrointestinal surgery, and gynecology. To the best of our knowledge, there have been no prior studies that examined lymphadenectomy across multiple departments within a single institution using a standardized dissection area based on a unified anatomical understanding.

Each published study to date has evaluated the number of lymph nodes using self-defined areas [5]. The clarification of this definition has significantly contributed to the detailed analysis of pelvic lymph-node regions. The PSPL-3 area mentioned in this paper falls into this exact category.

In the fields of urology, gastrointestinal surgery, and gynecology, there are important differences in lymph-node regions as a result of variations in the location of the primary tumor. In our institution, we observed the following trends. In rectal cancer, particular emphasis is placed on the internal iliac and obturator regions. Lymphadenectomy is performed with careful attention to the dorsal and more distal areas of the internal iliac vein. Dissection of the external iliac region is not routinely or aggressively pursued. In uterine cancer, emphasis is placed on the external iliac and obturator lymph-node regions. In bladder cancer and prostate cancer, lymph-node dissection is performed extensively in the external iliac, obturator, and internal iliac regions. This study enrolled high-risk prostate cancer patients who underwent lymph-node dissection, but low-risk patients were not enrolled. Lymph-node dissection is performed on a wide range of patients with bladder cancer, from those with lymph-node metastasis present preoperatively to those with non-muscle invasive bladder cancer. Whether pelvic lymph-node dissection is performed varies depending on the disease, and disease status may also affect the extent of the procedure. Therefore, it remains difficult to generalize across all cancer types.

The obturator lymph nodes are the region where lymphadenectomy is commonly emphasized across urology, gastrointestinal surgery, and gynecology. The importance of the dorsal region of the obturator nerve remains unclear. Several previous studies have examined lymphatic drainage and sentinel lymph nodes of pelvic cancers [6–11]. However, the localization of lymph nodes within the pelvis has not yet been sufficiently studied. In this study, dissection of the dorsal obturator nerve region did not increase the number of lymph nodes removed, but it did extend the surgical time by 15 min per side. This did not increase the positive detection rate. Previous reports on a small number of radical prostatectomy cases showed that dissection of the area dorsal to the obturator nerve increased the number of resected lymph nodes and positive detection rate [12]. Few papers refer to this dorsal region of the obturator nerve, because its definition has been vague, and it is a developing field. Future studies should analyze more cases and conduct more detailed evaluations, as well as perform large-scale verification.

In this study, we created a cross-disciplinary team across various departments, standardized the anatomical language, and conducted a prospective observational study on pelvic lymph-node dissection, after the dissection area was accurately defined. In cancer treatment, the quality of care can be improved by creating a team across multiple medical departments [13, 14]. While there are few similar studies in the field of surgery, extensive knowledge and skills within the pelvis are required for medical safety and surgery for advanced cancer cases. In this study, evaluation was performed by a single diagnostic pathologist. Several previous reports have documented the importance of central pathology review to reduce variability in diagnosis and staging and improve the reliability and reproducibility of data [15–18].

This study has several limitations, including bias in the registered diseases, particularly the limited number of rectal cancer cases. Furthermore, we were unable to investigate oncological benefits, such as recurrence and survival. QOL assessment reflects not only the impact of lymph-node dissection but also the effects of primary tumor resection. However, we conducted the first prospective pelvic lymph-node dissection study with detailed classification established in advance, using a central pathologist across pelvic surgery departments at the same institution. We plan to apply this approach for future cases of lymph-node dissection in the pelvis in patients with various diseases.

## Data Availability

The data presented in this study are available from the corresponding author upon reasonable request.
